# Trends in intellectual property rights protection for medical cannabis and related products

**DOI:** 10.1186/s42238-020-00057-7

**Published:** 2021-01-06

**Authors:** Joseph Wyse, Gilad Luria

**Affiliations:** Dr. Eyal Bressler & Co. Patent Attorneys, Lazrom House, 11 Tuval St., 5252226 Ramat-Gan, Israel

**Keywords:** Intellectual property rights, Medical cannabis, Marijuana, Cannabinoid, CBD, THC, Patents, Plant breeders’ rights, Nagoya Protocol, Access and benefit sharing

## Abstract

The purpose of this review is to advance the field of applied cannabis research by providing insights into the patenting of medical cannabis and current intellectual property rights (IPR) data.

Medical cannabis (MC) patent and plant breeders’ rights (PBR) registrations are filed on industrially applicable aspects of research. Studying the filing data and trends informs researchers of both gaps in current applied knowledge in MC (where patents have not been filed) and prior knowledge (where patents have already been filed).

Our focus is on those intellectual property rights (IPR) that are registered and germane to technical innovations in MC and related products. These are patents and PBR and thus exclude trade secrets, copyrights, franchises, or trademarks. Methods used for surveying the defined IPR landscape include searches of publicly available patent and PBR data and classifying the data according to the upstream–midstream–downstream innovation paradigm of the MC industry.

The findings suggest that the technical knowledge as expressed by patent filings is growing commensurate to the economic and legislative activity. Specific cannabis patents in agricultural technologies directed at improving yield, efficiency, and quality (known as “agritech”) are being filed and granted. These agritech-focused patents represent original novel and applied MC research achievements that address specific problems in cannabis cultivation, such as protection of the cannabis crop, maximizing cannabis yield, harvesting and post-harvesting of cannabis, and new advantageous varieties. Patents on *ex planta* and *in planta* cannabis genes expression have been published in recent years while patents on extraction methods for cannabinoids have increased since 2015. Much patent activity is in the downstream category of MC patient-oriented products and delivery systems for a very wide range of medical indications and disease conditions.

The emerging importance of access and benefit-sharing treaties and regulations is noted with implications on the industry briefly discussed. Patent data on leading and emerging patentee companies and institutions are shown. We also provide evidence of prior art and freedom to operate.

## Background

Medical cannabis (MC) or medical marijuana is defined herein as those products prescribed by physicians for patient therapy. There is no agreed definition of medicinal cannabis. The term is often used to refer to the therapeutic use of herbal cannabis and its constituents (Whiting et al. 2015). The cannabis plant contains more than 100 different chemicals, known as cannabinoids. Delta-9-tetrahydrocannabinol (THC) and cannabidiol (CBD) are the main relevant chemicals used in medicine (Bridgeman and Abazia 2017). THC produces the “high” that people feel when they smoke marijuana or eat foodstuffs containing it. The number of patients receiving medical cannabis products in the USA is estimated at over 3,000,000, with physicians in other countries such as the UK, Europe, Canada, Israel, and Australia increasingly prescribing MC to their patients (Number of Legal Medical Marijuana Patients - Medical Marijuana n.d.).

MC compounds emerge from processing beginning with a cultivated plant to a natural product (“plant-to-drug”), rather than a pharmaceutically synthesized molecule. Thus, the nascent MC industry has product pathways that sharply differ from the mainstream pharmaceutical industry.

MC-related matters are complicated in that, in almost all countries, cannabis is an illegal drug and is subject to legal constraints at the national and international level.

Although the geographical, historical, and cultural ubiquity of cannabis products has been described, knowledge surrounding the origins of cannabis use in folklore medicine is still vague (Heilig 2011; Zlas et al. 1993; Kuddus et al. 2013; Ren et al. 2019; West n.d.). In spite of the fact that cannabis has been used for centuries, the rigor demanded by present day regulatory authorities and modern medical standards raises particular challenges not often encountered in classical pharmaceutics based on defined single molecules.

These difficulties notwithstanding, MC companies must obtain governmental approval for specific indications if MC products are to enjoy the potential profits unlocked by manufacturing and distributing a novel approved medical product, influencing the scope and feasibility of cannabis drug development.

As MC use gains greater credibility, the market has come under constant and growing scrutiny from many stakeholders, including patient groups, physicians, research teams, companies, investors, government agencies, business analysts, lawyers, and Intellectual Property (IP) specialists. Much statistical information on MC is now routinely available and widely reported, some of which is introduced in this review.

The National Institutes of Health (NIH) reports 162 clinical trials on cannabis-based drugs overall (Search of: cannabis or cannabinoid or marijuana - List Results n.d.) with 58 clinical trials listed in the European Union Clinical Trials Register (Clinical Trials register - Search for Cannabis n.d.).

In 2018, the medical cannabis market was estimated at about 13.4 billion USD and is expected to reach 66.3 billion USD by 2025 with a compound annual growth rate (CAGR) of 22.9% from 2019 to 2025 (Medical Cannabis Market: Global Industry Trends, Share, Size, Growth, Opportunity and Forecast 2019-2024 n.d.). The North American Medical Marijuana index (founded in 2015) has already documented 49 companies with a total market capitalization of 32 billion USD (The Marijuana Index For Publicly Traded Companies n.d.). The worldwide market cap is $40.88 billion USD as of January 28, 2020 (Medical Marijuana Market Cap | MJNA n.d.).

As the increasing MC demand and economic and financial impact is contemplated, the importance of intellectual property rights (IPR) in this industry comes into focus. In this review article, the most important IPR instruments used in the MC industry are presented, accompanied by examples and data.

## Intellectual property rights

### The international framework for IPR

The World Trade Organization (WTO), founded in 1995, is the premier body regulating trade between nations and has enacted the Agreement on Trade-Related Aspects of Intellectual Property Rights (TRIPS). The TRIPS agreement provides benchmark standards for many types of intellectual property rights (IPR). IPR are those intangible rights owned and legally protected by a company or individual from outside use or implementation without consent. Intellectual property (IP) consists of patents, trade secrets, copyrights, know how, franchises, and trademarks. IPR are regarded as the transmission gear at the nexus of innovation, business, and law.

These types of IPR are most relevant to medical cannabis research:

#### Utility patents

Utility patents are an intellectual property right granted by a state within its territory to patent owner(s), excluding others from commercializing a technology recognized as novel and inventive claimed in the granted patent for a given time (up to 20 years with occasional judicial exceptions). Commercialization is defined as the commercial use, offer to use, sale, offer of sale, manufacture, or distribution of a patented technology or its products.

#### Plant breeders’ rights

IP instruments, known as Plant Variety Protection Rights (PVPR) or plant breeder’s rights (PBR), are an intellectual property right employed to protect commercialization of new varieties of cannabis plants developed by traditional breeding. A new cannabis variety unique in morphological characteristics may be protected using this IP instrument in most countries. To be eligible for a PBR, the new variety must be clearly distinguishable from any other commonly known variety and be sufficiently uniform and stable under cultivation.

#### Plant patents (USA)

An additional IPR protection for plant varieties, unique to the USA, termed a plant patent, exists. Plant patents extend the owner’s control and protection to asexual reproduction of a distinct and new variety of plant that expresses characteristics determined by its genotype. Plant patents can be granted for mutants (induced or spontaneous), hybrids, or transformed plants.

The exercise of IPR in the MC field incentivizes, accelerates, and rewards progress in this knowledge domain. Nations and regions all have their own particular histories and legacies entwined with ever changing social, economic, and cultural conditions. This influences local legislation on the scope and exploitation of IPR in MC.

Numerous articles have been published regarding cannabis plants and IPR but they are focused and localized to either a specific geographical territory (Rowand and Mcmahon 2018), a specific type of IPR (Jacobs 2017), specific fields in the cannabis plant industry (Flores-Sanchez and Ramos-Valdivia 2017; Gerra et al. 2010; Ranieri et al. 2016) or a specific species of Cannabis (Hahn 2019). Therefore, there is still a need for a broader analysis of IPR data concerning cannabis plants.

As the authors have noted, innovation in MC encompasses multiple activities in the journey from plant-to-drug. Therefore, it is not at all surprising that the relevant IPR instruments and policies are quite intricate, varied, and heterogenous, both regionally and nationally. In this study, we present IPR trends relevant to innovations in MC along the entire MC supply chain from upstream agritech (improved strains, genetic modification, plant material assessment, harvesting technology, and post-harvesting processing), midstream chemistry/analytics (extractions, purification methods, and separation methods), and downstream medical/biology (diseases, medical devices, compositions, formulations, and dosage forms). In this review, these categories will be applied. Furthermore, within this study, the researcher will be directed towards topics within IPR that bear consequences on MC research, including IPR-derived risks and limitations.

## Materials and methods

### Patent documents search

In order to find patent documents, the PatSnap search engine (https://www.patsnap.com/) was used. The search engine was instructed to return results from 116 national and regional databases (WIPO (WO)), Austria, Australia, Belgium, Benelux, Canada, Switzerland, Germany, Denmark, Spain, EUIPO, Finland, France, Great Britain, China Hong Kong, Ireland, Israel, India, China Macao, Netherlands, Norway, New Zealand, Poland, Russia, Sweden, Singapore, Thailand, China Taiwan, United Arab Emirates, Armenia, ARIPO, Argentina, Bosnia and Herzegovina, Bulgaria, Bahrain, Brunei Darussalam, Bolivia, Brazil, Belarus, Chile, Colombia, Costa Rica, Czech Slovak Rep., Cuba, Cyprus, Czech Republic, East Germany, Dominica Rep., Algeria, EAPO, Ecuador, Estonia, Egypt, GCC, Georgia, Greece, Guatemala, Honduras, Croatia, Hungary, Indonesia, Iceland, Italy, Jordan, Kenya, Kyrgyzstan, Cambodia, Kazakhstan, Lao People’s Democratic Republic, Lebanon, Lithuania, Luxembourg, Latvia, Morocco, Monaco, Moldova, Montenegro, The Republic of North Macedonia, Mongolia, Malta, Malawi, Mexico, Malaysia, Nicaragua, OAPI, Panama, Peru, Philippines, Portugal, Paraguay, Romania, Republic of Serbia, Saudi Arabia, Slovenia, Slovakia, San Marino, Soviet Union, El Salvador, Tajikistan, Tunisia, Turkey, Trinidad and Tobago, Ukraine, Uruguay, Uzbekistan, Venezuela, Vietnam, Yugoslavia, South Africa, Zambia, and Zimbabwe.

The IP searches mentioned in this publication were carried out as follows:

**Table:** 

Search	Searched publication section	Keywords used	Comments
**Search A**: General search	Independent claims	(cannab* OR marijuana) combined with International Patent Classification code A61	A61—medical or veterinary science; hygiene
**Search B**: MC upstream technologies	Independent claims	(cannab* OR marijuana) AND (cultivat* OR soil OR light OR water OR irrigat* OR planting OR selecting OR harvest OR post harvest OR packing OR storing OR storage OR curing OR drying OR pick OR pest OR herb OR ripen) combined with the International Patent Classification code A01 (excluding the A01H category which is *inter alia,* directed to *ex planta* technology).	A patent or patent application was deemed a specific cannabis or marijuana upstream technology patent if the terms “cannabis or marijuana” appeared in the independent claim to the exclusion of other named plant species. 236 patent documents were returned by the search engine concerning the specific field of upstream MC technologies, representing 101 patent families. Manual inspection was carried out of at least one independent claim of each of the 101 patent families. 39 patents or patent applications were deemed, by the above criteria, to be specific MC upstream technology patents.
**Search C**: MC upstream technologies	Independent claims	(cannabinoid OR cannabis OR marijuana) AND (sensor OR sensing) AND (plant OR biomass OR harvest OR field OR greenhouse or hothouse)	A patent or patent application was deemed a specific cannabis or marijuana upstream technology patent if the terms “cannabis or marijuana” appeared in the independent claim to the exclusion of other named plant species, and sensor or sensing, and plant or biomass or harvest or field or greenhouse or hothouse. Manual inspection was carried out of at least one independent claim of each of the 26 patent families. 6 patent families were identified in this way. Each was then compared to the results of the first search in order to eliminate double counting.
**Search D**: MC upstream technologies—disease control	Title and abstract	(cannabis OR marijuana) combined with International Patent Classification codes: (A01N OR A01N25 OR A01P OR A01P7).	A01N—preservation of bodies of humans or animals or plants or parts thereof A01N25—biocides, pest repellants or attractants, or plant growth regulators, characterized by their forms, or by their non-active ingredients or by their methods of application. A01P—biocidal, pest repellant, pest attractant, or plant growth regulatory activity of chemical compounds or preparations A01P7—arthropodicides
**Search E**: MC upstream technologies—disease control	Independent claims	(cannabis OR marijuana) combined with International Patent Classification codes: (A01N OR A01N25 OR A01P OR A01P7).	A01N—preservation of bodies of humans or animals or plants or parts thereof A01N25—biocides, pest repellants or attractants, or plant growth regulators, characterized by their forms, or by their non-active ingredients or by their methods of application A01P—biocidal, pest repellant, pest attractant, or plant growth regulatory activity of chemical compounds or preparations A01P7—arthropodicides
**Search F**: MC upstream technologies—*in planta* genetic modification technologies	Title and abstract	(cannabinoid AND (recombinant OR recomb* OR construct OR promoter OR vector OR genetically modified OR gm OR plasmid OR gene insertion OR insert OR gene editing OR crispr OR talens) OR (expression of cannabinoid) OR (biosynthesis of cannabinoid) combined with International Patent Classification codes: (C12N and/or A01H) for at least 4 iterations of IPC.	C12N–M or enzymes, compositions thereof; propagating, preserving, or maintaining microorganisms; mutation or genetic engineering; culture media A01H—new plants or processes for obtaining them; plant reproduction by tissue culture techniques
**Search G**: MC midstream technologies	Independent claims	(cannabis OR marijuana) AND (extract* OR purif* OR separat* OR preserv*) AND (supercri* OR ultra* OR solvent).	
**Search H**: MC downstream technologies	Independent claims	cannab* OR marijuana) AND (Alzheimer’s disease or appetite loss or cancer or Crohn’s disease OR celiac disease OR eating disorders OR anorexia OR bulimia nervosa OR epilepsy OR glaucoma OR anxiety OR schizophrenia OR posttraumatic stress disorder OR multiple sclerosis OR Parkinson’s disease OR tremor OR muscle spasms OR tooth decay OR dental caries OR nausea OR vomit* OR pain OR burns OR psoriasis OR cachexia OR inflammation) AND (composition OR compound OR formulation) AND (disease OR condition OR treatment OR therapy OR medic*) NOT synthetic) combined with International Patent Classification codes: A61	A61—medical or veterinary science; hygiene

### PBR search

In order to find registrations of plant breeders’ rights, the Community Plant Variety Office (CPVO) database was used (https://cpvo.europa.eu/en). Under “variety finder,” *Cannabis sativa* was searched under the species Latin name. Only PBRs were selected with all types of applications being searched (including rejected, examined, and expired applications).

### Generation of graphs, tables, and figures

PatSnap’s Insights analytical tool was used to generate patent graphics of filing rates, geographic territories, and assignee analysis. The Insights tool processes patent data to produce graphs and figures. The insights database selection tool is configured to inspect the 116 available databases listed above. Raw patent data was filtered by key word selection and International Patent Classification (IPC) selection. The search for most cited patent applications in the field of MC downstream technologies (Table 7) was based on the results of search H, with the table manually generated from data obtained by the Insights analytical tool.

## Results

In order to search patent documents presented in this publication, the authors used the PatSnap search engine (see the “Materials and methods” section). Using broad keywords, approximately 2000 patent families directed to various MC technologies were found (see the “Materials and methods” section; search A).

**Fig. 1:**
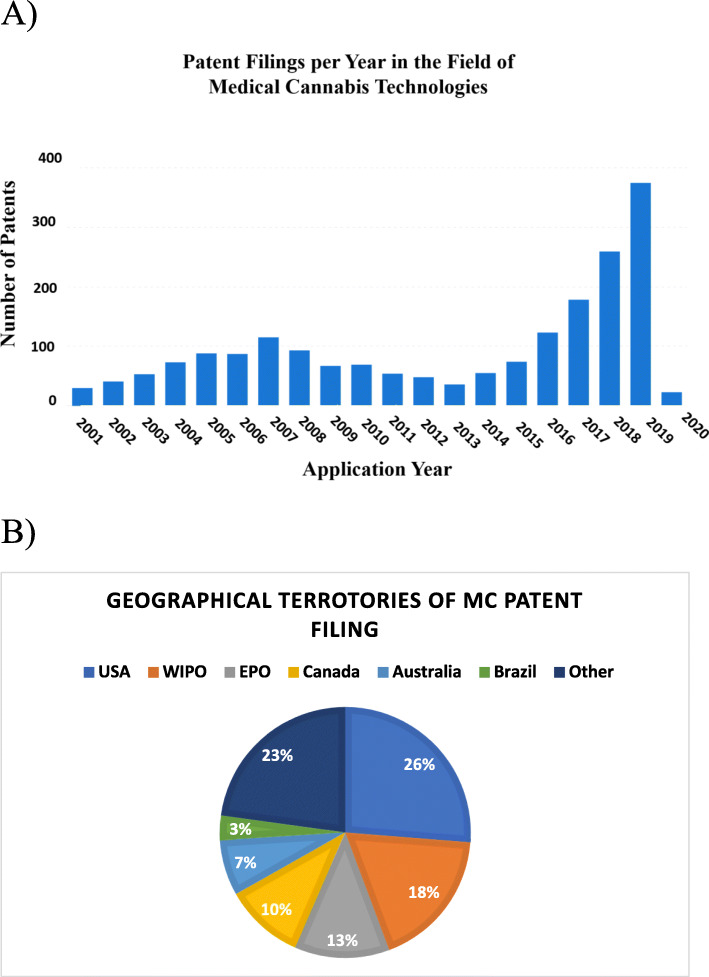
Overview on utility patents in the field of medical cannabis. **a** Graphically depicts the patent filing rate from 2001 to 2020. The total number of medical cannabis-related utility patents is represented in each column. **b** Graphical depiction of the main countries in which the MC-related utility patents were filed. The figures were generated using “Insights,” an analytic tool by Patsnap (https://www.patsnap.com). For more information on the PatSnap software operations, see the “Materials and methods” section

Figure 1a shows the patent family filing rate rose over 6-fold, from approximately 60 filings in 2013 to 380 filings as of June 2020. The filings are concentrated in the USA (26.25%), Europe (12.26%), Canada (10.25%), and Australia (7.20%) with the remainder filed in other territories or at the intermediate international PCT (Patent Cooperation Treaty; WIPO) stage (Fig. 1b).

### Upstream MC technologies

Upstream technologies are an important differentiator from traditional Pharma IP domains as these technologies are centered upon agricultural innovation. Before a cannabis product can be produced or even investigated as a medical candidate, strains must be selected, followed by the development and implementation of efficient cultivation methods. Companies that can repeatedly deliver high-quality harvests of appropriate plants with predictable yields and cannabinoid content will be at an advantage and protect their beneficial applied research by means of registered IPRs, namely plant breeders rights, plant patents, and utility patents.

Traditional breeding is increasing the production of Cannabis plants with unique traits. intellectual property rights to such plants are typically registered as PBR.

In order to characterize the cannabis-related PBR filings, a search was conducted for PBR registrations pertinent to *Cannabis sativa* (see “Materials and methods” section). Data obtained from the Community Plant Variety Office (CPVO) database, which includes the global figures, revealed 434 *Cannabis sativa* varieties applications. PBR filed in the European Union are the most numerous (48%), followed by Australia (8%), Russia (7%), and Canada (5.7%). The USA only has 4 listings, all approved in 2019. This is probably because, in the USA, it is only since 2019 that plant variety protection certificates under the International Union for the Protection of New Varieties of Plants (UPOV) are obtainable for new and distinct *Cannabis sativa* varieties with THC content not exceeding 0.3%.

As for plant patents, 30 cannabis plant patents and applications have been filed in the USA. The first cannabis plant patent USPP27475 (Cannabis plant named “Ecuadorian Sativa” (USPP27475P2 - Cannabis plant named ‘Ecuadorian Sativa’ - Google Patents n.d.)) was granted in 2016 to Kubby Patent and Licenses LLC. Recently, the Biotech Institute LLC was granted US Plant Patent USPP31535 for their “Lemon Crush OG” (USPP31535P3 - Cannabis plant named ‘LEMON CRUSH OG’ - Google Patents n.d.) cultivar.

From the data revealed in the above searches, it can be seen that researchers are exploiting the relatively low technological barrier needed to qualify for PBR or Plant Patent protection. They do this by protecting varieties for morphological trait uniqueness (as required by the authorities), while in practice protecting the therapeutic or pharmacological value of their plant variety.

As for MC utility patents focused on upstream technologies, 236 patent documents were found (see the “Materials and methods” section; search B), representing 101 patent families. Following a manual inspection, 39 patents or patent applications were deemed by the criteria described in the “Materials and methods” section to be specific MC upstream technology patents.

To cover additional areas in this specific field, mainly sensing monitoring in greenhouses, a second search was carried out (see the “Materials and methods” section; search C). Fifty-two patent documents were returned by the search engine, representing 26 patent families. Following a manual screening, 4 patent families were identified. Overall, it was determined that there were 43 independent upstream specific MC upstream technology patents.

The MC upstream technology patent data reveals IPR activity in the following areas: seed and crop protection, cultivation methods and equipment, precise crop harvesting in situ, post-harvest methods, genetic engineering technologies, and disease and pest control.

Representative patent applications of the first four topics are described in Table 1.

**Table 1:** Patents on medical cannabis upstream technologies (growth and harvesting)

	Upstream technology topic	
	**Seed and crop protection**	
**Title**	**Short description**	**Patent doc. number**
Rhamnolipid repellent and disinfectant (WO2020102800A1 - Rhamnolipid repellent and disinfectant - Google Patents n.d.)	Coating cannabis seeds with rhamnolipid to prevent pathogens	WO2020102800A1
	**Cultivation methods and equipment**	
**Title**	**Short description**	**Patent doc. number**
Plant artificial seeds having multilayers and methods for the production thereof (WO2013096536A1 - Plant artificial seeds having multilayers and methods for the production thereof - Google Patents n.d.)	Artificial seed in dedicated container	WO2013096536A
Method of hydroponic growing of plants (US9822042B2 - Method of hydroponically growing of plants - Google Patents n.d.)	Hydroponically cultivating cannabis plants, controlled nutrient release	US9822042
Polymeric film (WO2020078766A1 - Polymeric film - Google Patents n.d.)	Polymeric greenhouse film for enhancing cannabis plant growth by blocking appropriate light spectra	WO2020078766A1
Cannabis growth methods and systems (US10021838B1 - Cannabis growth methods and systems - Google Patents n.d.)	Growth of cannabis in small scale environmentally controlled cabinets	US10021838B1
	**Precise crop harvesting**	
**Title**	**Short description**	**Patent doc. number**
Optical determination of cannabis harvest date (WO2020084391A1 - Optical determination of cannabis harvest date - Google Patents n.d.)	Sensing systems measuring cannabinoids in cannabis plants	WO2020084391A1
Devices, systems and methods of identifying plants, plant material and plant state (WO2019237203A1 - Dispositifs, systèmes et procédés d’identification de plantes, de matériel végétal et d’état végétal - Google Patents n.d.)	Sensing cannabis plant state via gas sensors in the ambient air surrounding the plant	WO2019237203A1
	**Post-harvest methods**	
**Title**	**Short description**	**Patent doc. number**
Method and apparatus for harvesting trichomes from cannabis plants (CA3050648A1 - Method and apparatus for harvesting trichomes from cannabis plants - Google Patents n.d.)	Harvesting trichomes from frozen cannabis plant biomass	CA3050648A1

Table 1 indicates 4 patents specifically directed to cannabis cultivation although most of the patents in the cultivation and equipment area are controlled growth and seed technologies applicable to many plant species, including cannabis. Two patents were identified directed to harvesting methods for cannabis plants in situ.

An additional patent search was carried out specifically for controlling diseases and pests to which the cannabis plant grown as a crop is particularly prone (see the “Materials and methods” section; search D). This search results in 131 patent documents, grouped into 86 patent families. Several examples for patent documents concerning controlling plant diseases, including cannabis, are detailed and shown in Table 2.

**Table 2:** Patents on medical cannabis upstream technologies (disease and pest control)

Title	Short description	Patent doc. number
Systems and methods for indoor plant cultivation, storage, and pest control (US20180325036A1 - Systems and methods for indoor plant cultivation, storage, and pest control - Google Patents n.d.)	A method for controlling pests for indoor cannabis applications using a regime of oxygen and CO_2_ exposure at normal temperatures	US20180325036A1
Method of treating marijuana plants with a reactive oxygen species (WO2016095024A1 - Method of treating marijuana plants with a reactive oxygen species - Google Patents n.d.)	A pesticide application is applied to field grown cannabis plants using compounds containing reactive oxygen, such as peroxides and ozone	WO2016095024A1

When a further, more limiting search was carried out in the context of disease control in cannabis plants, this time including cannabis or marijuana in “independent claims” and not in the “Title and abstract” of the patent documents (see the “Materials and methods” section; search E), 21 documents were retrieved. Manual inspection of the relevant texts revealed that these were patents providing solutions targeting a number of plant species, cannabis merely being among the claimed species.

From these data, MC researchers may note that protecting and controlling cannabis plant diseases by biological or eco-friendly non-hazardous compounds or materials would appear to be an area worth innovating and patenting in order to achieve cannabis-specific patentable technology and enhance the MC infrastructure.

A major finding of this present study is the large amount of patent data on *ex planta* (Table 3) and *in planta* (Table 4) genetic modification technologies (see search F in the “Materials and methods” section), including production of cannabinoids from microorganisms, suspended plant cells, and recombinant DNA technologies (genetic modification, expression of new genes, gene silencing, gene editing, new trait selection methods, and novel breeding processes). As more MC products of genetically manipulated origin becomes available, it is likely to influence the perception of MC in some circles as a natural herbal product.

**Table 3:** Patents on medical cannabis upstream technologies (*ex planta* genetic engineering)

Title	Short description	Patent doc. number
Chemical engineering processes and apparatus for the synthesis of compounds (US20200048664A1 - Chemical engineering processes and apparatus for the synthesis of compounds - Google Patents n.d.)	Methods and a system for producing cannabinoids and cannabinoid analogs via enzymatic reactions	US20200048664A1
Bioenzymatic synthesis of THC-v, CBV and CBN and their use as therapeutic agents (US10538790B2 - Bioenzymatic synthesis of THC-v, CBV and CBN and their use as therapeutic agents - Google Patents n.d.)	Methods for bio-enzymatic synthesizing of THC-v, CBV, and CBN	US10538790
Biosynthesis of cannabinoid prodrugs and their use as therapeutic agents (US20190382814A1 - Biosynthesis of cannabinoid prodrugs and their use as therapeutic agents - Google Patents n.d.)	Methods for producing cannabinoid prodrugs and a system for the large-scale production of the prodrugs	US20190382814A1
Apparatus, methods and composition for synthesis of cannabinoid compounds (US20190382708A1 - Apparatus, methods and composition for synthesis of cannabinoid compounds - Google Patents n.d.)	Systems and methods for producing a cannabinoid product with cannabinoid synthase in an immiscible phase	US20190382708A1
Microorganisms and methods for the fermentation of cannabinoids (WO2019071000A1 - Microorganisms and methods for the fermentation of cannabinoids - Google Patents n.d.)	Methods for synthesizing cannabigerolic acid (CBGA) and other cannabinoids in bacterial systems	WO2019071000A1
Production of cannabinoids in yeast (WO2019014490A1 - Production of cannabinoids in yeast - Google Patents n.d.)	Genetically modified yeast with GPP pathway genes, olivetolic acid producing genes, cannabinoid precursor or cannabinoid producing genes, or hexanoyl-CoA-producing genes	WO2019014490A1
Generation of water-soluble cannabinoid compounds in yeast and plant cell suspension cultures and compositions of matter (US20190078168A1 - Generation of Water-Soluble Cannabinoid Compounds in Yeast and Plant Cell Suspension Cultures and Compositions of Matter - Google Patents n.d.)	Systems, methods, and compositions for the generation of water-soluble cannabinoids in yeast, and other plant cell suspension cultures	US20190078168A1
Microbial engineering for the production of cannabinoids and cannabinoid precursors (WO2017139496A1 - Microbial engineering for the production of cannabinoids and cannabinoid precursors - Google Patents n.d.)	Compositions and methods for producing cannabinoids and cannabinoid precursors in microorganisms from a carbohydrate source	WO2017139496A1
Production of cannabinoids in microorganisms from a carbon sugar precursor (US20190382813A1 - Production of cannabinoids in microorganisms from a carbon sugar precursor - Google Patents n.d.)	A method for biosynthetic production of cannabinoids in microorganisms from a carbon source precursor	US20190382813A1
Biological composition based on engineered *Lactobacillus paracasei* subsp. *paracasei* f19 for the biosynthesis of cannabinoids (EP3067058A1 - Biological composition based on engineered lactobacillus paracasei subsp. paracasei f19 for the biosynthesis of cannabinoids - Google Patents n.d.)	A method for producing therapeutic cannabinoids characterized by administering to a host the probiotic *Lactobacillus Paracasei* subsp. *Paracasei* F19, genetically modified to produce and secrete cannabinoids from *Cannabis sativa*	EP3067058A1
Cannabinoid production in algae (WO2019202510A1 - Cannabinoid production in algae - Google Patents n.d.)	A system and method for producing a cannabinoid in genetically modified algae expressing several metabolic enzymes	WO2019202510A1

**Table 4:** Patents on medical cannabis upstream technologies (*in planta* genetic engineering)

Title	Short description	Patent doc. number
Plants and methods for increasing and decreasing synthesis of cannabinoids (US20200181631A1 - Plants and methods for increasing and decreasing synthesis of cannabinoids - Google Patents n.d.)	CRISPR/Cas9 system gene editing system to alter the expression of cannabinoids in plants	US20200181631A
Modulation of cannabinoid profile in cannabis (WO2020035869A1 - Modulation of cannabinoid profile in cannabis - Google Patents n.d.)	Gene editing technology to modify metabolite pathways in the cannabis plant or cells	WO2020035869A1
Systems and methods for enhancing trichome formation and density in cannabis (US20190352662A1 - Systems and methods for enhancing trichome formation and density in cannabis - Google Patents n.d.)	Insertion of heterologous genetic material to create a plant with upregulated trichome formation	US20190352662A1
Trichome specific promoters for the manipulation of cannabinoids and other compounds in glandular trichomes (US20190225975A1 - Trichome specific promoters for the manipulation of cannabinoids and other compounds in grandular trichomes - Google Patents n.d.)	Glandular trichomes of the cannabis plants are genetically engineered to produce cannabinoids in *Cannabis sativa*, and also in *Nicotiana. tabacum*	US20190225975A1

In conclusion, the topics in this particular field vary but there appears to be room for research intervention upstream because absolute numbers of patents are low and many of the patents are not specific to particular cannabis-related problems. Additionally, new strains, traits, and genetic manipulations and editing are advancing, which may affect the discourse around MC.

### Midstream technologies

The major patenting activity in this part of the supply chain is still in natural product technology, namely extraction of cannabinoids, terpenes, and flavonoids as well as purification, separation, and preservation methods. The challenge here is to provide easily scalable extraction methods with a high yield of the desired compounds, chiefly the non-psychotropic ingredient CBD. Other potentially medically useful compounds, referred to as “entourage” compounds, are present in low concentrations in the natural state and require the development of meticulous extraction, separation, and purification techniques. A specialized patent search was performed as described in the “Materials and methods” section (search G).

**Fig. 2:**
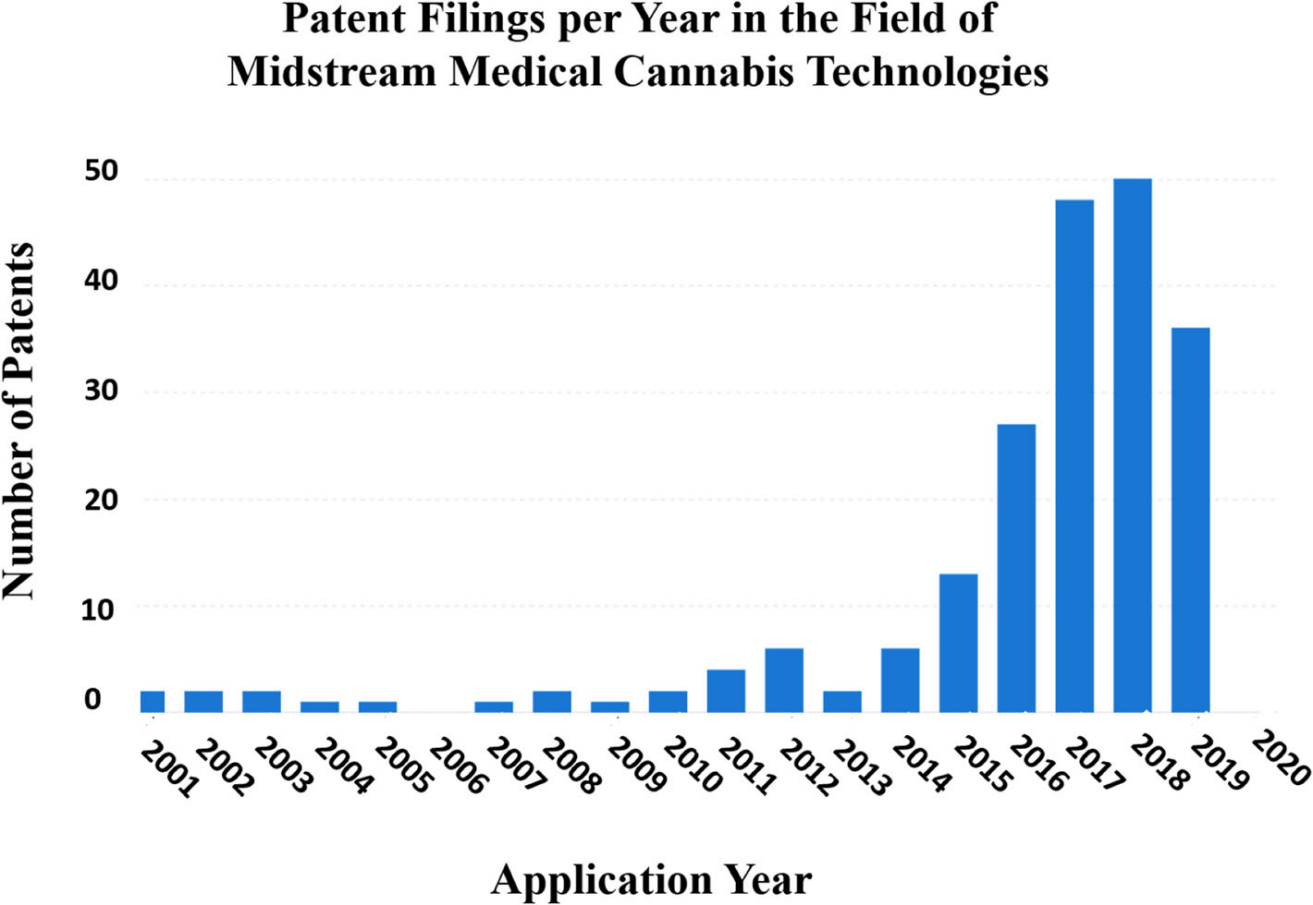
Patent filing trend in the field of midstream medical cannabis technologies. Graphical depiction of the number of patents concerning midstream medical cannabis technologies filed from 2001 to 2020. The figure was generated using “Insights,” an analytic tool by PatSnap (https://www.patsnap.com). For more information on the PatSnap software operations, see the “Materials and methods” section

Figure 2 illustrates over 200 filings in this field since 2015 whereas, prior to that year, the commercial (and patenting) interest in extraction methods from cannabis plants was close to negligible. There are several extraction methods being patented, including ultrasonic disruption, electroporation, and organic solvent extractions, but since 2013, the patent activity has been mainly dominated by supercritical gas extraction methods. GW Pharmaceuticals, a market leader in medical cannabis technologies, is a holder of several patents in this latter technology. Table 5 presents a few examples for such patent applications.

**Table 5:** Patents on medical cannabis midstream technologies

Title	Short description	Patent doc. number
Processes and apparatus for extraction of active substances and enriched extracts from natural products (US9034395B2 - Processes and apparatus for extraction of active substances and enriched extracts from natural products - Google Patents n.d.)	Processes for preparing purified extracts from crude extracts of natural products, by extraction with hot gas	US9034395
Extraction of pharmaceutically active components from plant materials (US20040033280A1 - Extraction of pharmaceutically active components from plant materials - Google Patents n.d.)	A method for preparing a botanical drug substance for incorporation in to a medicament. The medicament comprises cannabinoids obtained by extraction from cannabis	US20040033280A1

### Downstream technologies

Medical cannabis extractions obtained from midstream technologies are formed into compositions, formulations, and compounds for which patent protection is sought in regard to the treatment of a number of medical conditions. These indications have been associated with MC treatment and include Alzheimer’s disease; appetite loss; cancer; Crohn’s disease; celiac disease; eating disorders such as anorexia and bulimia nervosa; epilepsy; glaucoma; mental health conditions such as anxiety, schizophrenia, and posttraumatic stress disorder; multiple sclerosis; Parkinson’s disease; tremor; muscle spasms; tooth decay or dental caries; nausea and vomiting caused by chemotherapy; pain; burns; psoriasis; cachexia; and inflammation. Therefore, a patent search for MC downstream technologies related specifically to the above medical conditions was carried out (see “Materials and methods” section, search H).

**Fig. 3:**
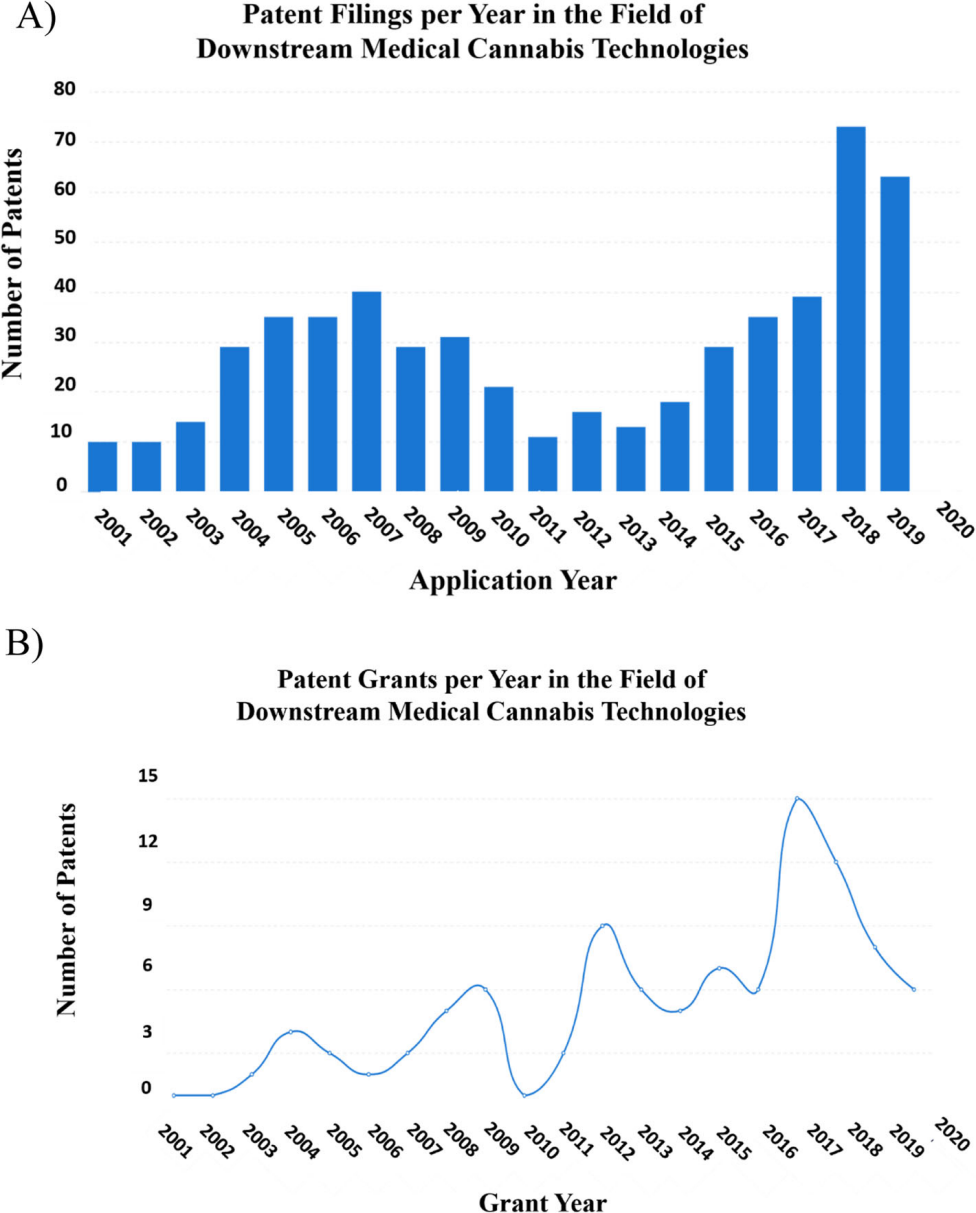
Patent filing trend in the field of downstream medical cannabis technologies. **a** Graphical depiction of the number of patents concerning downstream medical cannabis technologies filed from 2001 to 2020. **b** Graphical depiction of the number of patents that were granted through 2001–2020. The figures were generated using “Insights,” an analytic tool by PatSnap (https://www.patsnap.com). For more information on the PatSnap software operations, see the “Materials and methods” section

Approximately 570 patent families (2200 patent documents) have been filed in these downstream technologies, with the filing rate rising steadily since 2011–2013 (Fig. 3a). The steep increase in patent filing and grants (Fig. 3a and b) since 2011–2013 is consistent with the recognition by industry that the number of US states allowing legal medical cannabis was reaching a critical number. In many other countries, by this time, legalization and medical opinion had progressed towards the medicalization of cannabis and the liberalization of its use.

**Fig. 4:**
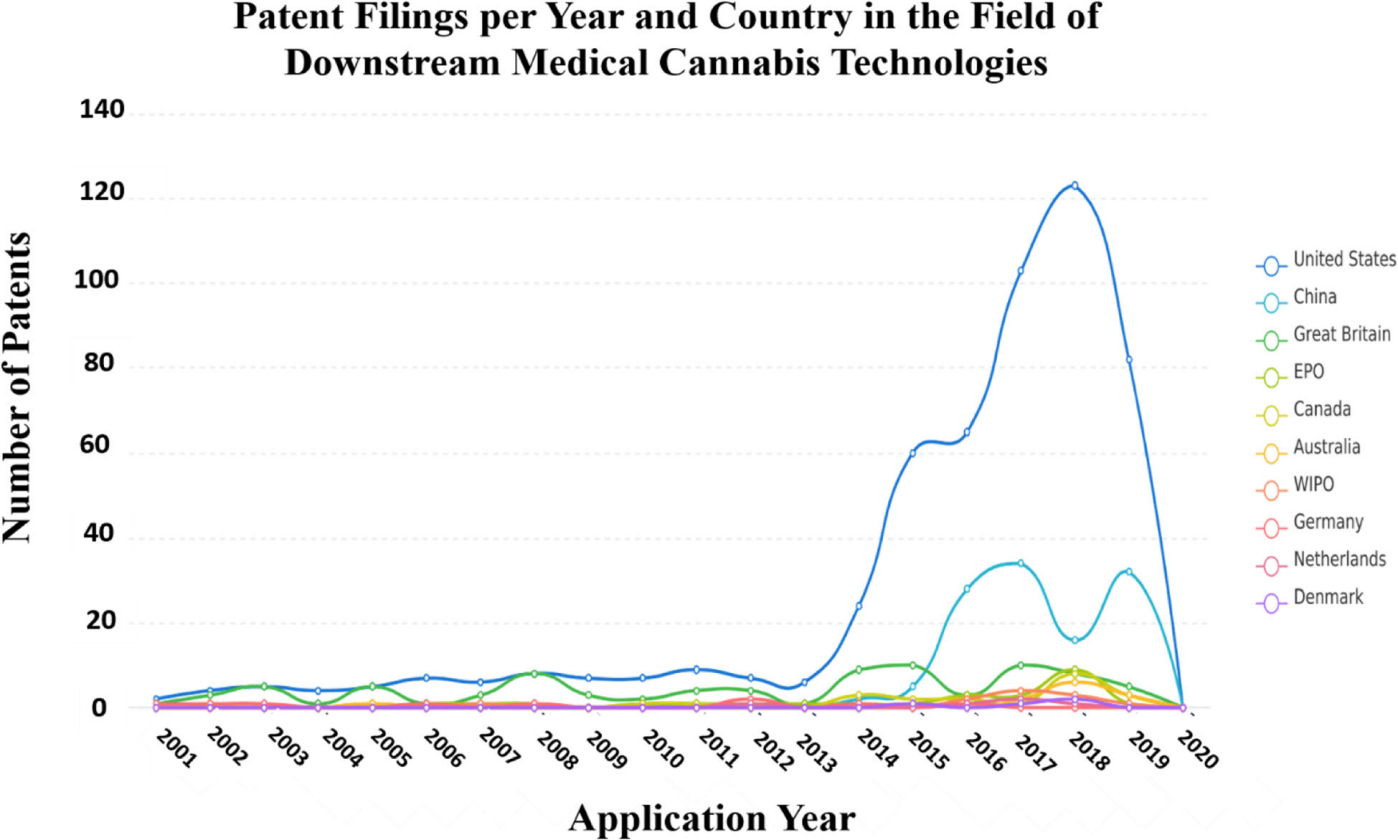
Main countries in which patents related to downstream medical cannabis technologies were filed. Graphical depiction of the number of patents concerning downstream medical cannabis technologies filed from 2001 to 2020 and the main countries in which these patents were filed. The figure was generated using “Insights,” an analytic tool by PatSnap (https://www.patsnap.com). For more information on the PatSnap software operations, see the “Materials and methods” section

Additionally, it can be seen in Fig. 4, which depicts the national and regional patent filing trends in the field of MC downstream innovations, that the USA, followed by China and the European countries, outstrip all other territories as a market and originator of these technologies.

**Fig. 5:**
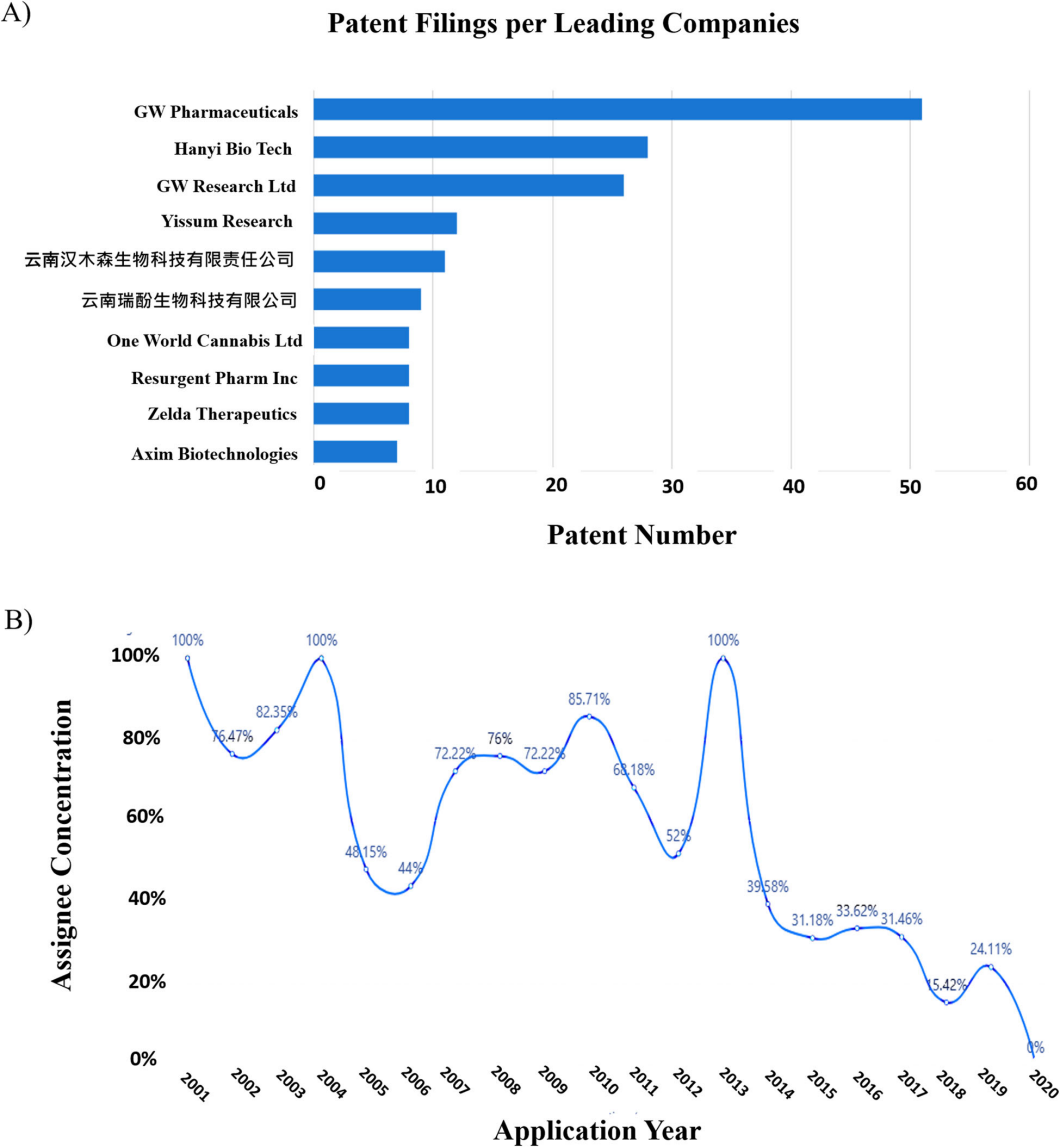
Main patent assignees and markets of patents related to downstream medical cannabis technologies were filed. **a** Graphical depiction of the main assignees in the field of downstream medical cannabis technologies and the number of patent applications they own. **b** Graphical depiction the concentration of the main patent assignees from 2001 to 2020. The figures were generated using “Insights,” an analytic tool by PatSnap (https://www.patsnap.com). For more information on the PatSnap software operations, see the “Materials and methods” section

In industries driven by innovation, it is crucial for companies to continuously construct and defend a patent portfolio commensurate with the core proprietary technologies upon which their commercial well-being depends. The company patent portfolio should also include technologies that may be contemplated by their competitors so as to exclude them from the market, at least for a time. Figure 5a lists the top 10 patent assignees in the field of downstream MC technologies. GW Pharmaceuticals is a company, which on the evidence of their patent filing, market, and financial data implements patent strategies successfully. It will be noted that Yissum Research development is the technology transfer company of the Hebrew University of Jerusalem, Israel. So, their motivation for holding a substantial patent portfolio relevant to medical cannabis is for licensing purposes and/or for supporting tech incubator companies. Other companies, such as Zelda Therapeutics (currently known as Zelira Therapeutics Ltd), Cannabics Pharma, and Axim® Biotechnologies Inc., will find their patent portfolios tempting to future partners and investors or serving as assets that may be sold or licensed for royalties.

The most dominant assignee is GW Pharmaceuticals and its subsidiary GW Research, which together hold about 80 patents in this MC-related field. Interestingly, we can observe that, in spite of GW’s ownership of a large group of patents, the overall patent monopolization in the field has been on a downward trend. This is signified by the decreased ratio of the number of patents of the top 10 assignees relative to the total number of applications when compared to the ratio found in the early 2000s (Fig. 5b). This observation is a characteristic of a nascent industry and is evidence of the rapid formation of new companies and startups in recent years creating their own patent eligible technologies.

Moreover, in the last 5 years (2016–2020), companies such as Buzzelet (presently Bazelet) and Radient Technologies have been resurfaced and also filed patent applications. Such companies provide a burgeoning source of new innovation and monetizable patents. They are often regarded as potential partners to companies with complementary technologies or acquisition targets for bigger companies or competitors.

An analysis of the origin and patent protection of the technology field in Europe, China, Japan, Korea, and the USA reveals that companies typically seek to protect their inventions in their own local market and are possibly overlooking potentially profitable markets elsewhere as legislation continues to liberalize.

### Innovations directed to medical conditions

An obvious trend observed among the resulting patent literature was related to MC modes of delivery. Various modes of delivery are used and patented to administer cannabinoid formulations and compositions to the patient, such as transdermal, topical, oral, buccal, sublingual, and rectal. Medical cannabis compounds may be smoked or inhaled. Medical cannabis can be in edible form, capsules, tablets, pills, ampoules, oils, waxes, liposomes, particles, suspensions, cartridges, droppers, micelles, pastes, and more. Table 6 depicts several patent applications in the field of downstream MC for the treatment of various medical conditions in humans and in other animals.

**Table 6:** Patents on medical cannabis downstream technologies

Title	Short description	Patent doc. number
Suppositories comprising cannabinoids (US10543190B2 - Suppositories comprising cannabinoids - Google Patents n.d.)	A suppository composition comprising cannabinoids enabling easy absorption through mucosal membrane for patients who have difficulties swallowing and need cannabinoid treatment for pain, nausea, or inflammatory bowel diseases	US10543190
Parenteral formulations (US20190314296A1 - Parenteral formulations - Google Patents n.d.)	Parenteral cannabinoid formulations, and more particularly, cannabinoid containing intravenous (IV) formulations for intravenous administration	US20190314296A1
Cannabinoid compositions, methods of manufacture, and use thereof (US20190060381A1 - Cannabinoid compositions, methods of manufacture and use thereof - Google Patents n.d.)	Oral compositions for the treatment of cancer-related cachexia and anorexia syndrome (CACS) in a combined immediate release and sustained release cannabinoid delivery system	US20190060381A1
Composition containing cannabidiol and/or cannabidivarin and application of composition in treatment of dysmenorrhea (WO2019052303A1 - Composition containing cannabidiol and/or cannabidivarin and application of composition in treatment of dysmenorrhea - Google Patents n.d.)	Transdermal cannabidiol and cannabidivarin compositions for preventing and/or treating women's dysmenorrhea, and these compositions and can be applied with female hygiene products	WO2019052303A1
Compositions and methods for treating cancer (US20200030282A1 - Compositions and methods for treating cancer - Google Patents n.d.)	Compositions and therapeutic methods for treating cancers by means of defined fractions of cannabis plant extracts	US20200030282A1
Composition and methods for production and use (US20180344662A1 - Veterinary composition and methods for production and use - Google Patents n.d.)	A method for treating bees’ viruses and protect them from colony collapse disorder	US20180344662A1

All of the patents and patent applications mentioned above, as well as the rest of the data mentioned in this article, are at various stages of the patent process, chiefly examination or grant. In order for a patent to proceed to grant, the patent examiner must be satisfied that the patent application is new and inventive. The examiner will endeavor to provide “prior art” (publicly available literature published before the date of the patent application) citations to challenge the novelty and inventive step of the patent application. Table 7 provides data on patents most cited by patent examiners during examinations of subsequent patent applications relevant to the application in question. Such patents therefore represent prior art and may be considered barriers to patentability, possibly even restricting “freedom to operate,” the legal operating space available to researchers which is unoccupied by pre-existing valid patents.

**Table 7:** Patents on medical cannabis most cited by patent examiners

Title and patent or patent application no.	Main topic	Assignee	Priority date	Citations
US6403126 Cannabinoid extraction method (US6403126B1 - Cannabinoid extraction method - Google Patents n.d.)	Extraction	WEBSAR INNOVATIONS	2001	160
US20020111377 Transdermal delivery of cannabinoids (US20020111377A1 - Transdermal delivery of cannabinoids - Google Patents n.d.)	Delivery mode	KENTUCKY ECONOMIC DEV FINANCE AUTHORITY	2001	91
US20110052694A1 Use of cannabidiol prodrugs in topical and transdermal administration with microneedles (US20110052694A1 - Use of cannabidiol prodrugs in topical and transdermal administration with microneedles - Google Patents n.d.)	Delivery mode	ZYNERBA PHARM	2010	90
WO2009007697A1 New pharmaceutical formulation comprising cannabidiol and tetrahydrocannabidivarin (WO2009007697A1 - New pharmaceutical formulation comprising cannabidiol and tetrahydrocannabidivarin - Google Patents n.d.)	Formulation	GW Pharma	2008	70
US20040049059A1 Method for producing an extract from cannabis plant matter, containing a tetrahydrocannabinol and a cannabidiol and cannabis extracts (US20040049059A1 - Method for producing an extract from cannabis plant matter, containing a tetrahydrocannabinol and a cannabidiol and cannabis extracts - Google Patents n.d.)	Extraction	BIONORICA ETHICS GmbH	2004	73
US6113940 Cannabinoid patch and method for cannabis transdermal delivery (US6113940A - Cannabinoid patch and method for cannabis transdermal delivery - Google Patents n.d.)	Delivery mode	PATCHTEK INC	1997	68
US6949582 Method of relieving analgesia and reducing inflammation using a cannabinoid delivery topical liniment (US6949582B1 - Method of relieving analgesia and reducing inflamation using a cannabinoid delivery topical liniment - Google Patents n.d.)	Delivery mode and treatment	WALTER H WALLACE	2005	72
US20060039959 Film-shaped mucoadhesive administration forms for administering cannabis agents (US20060039959A1 - Film-shaped mucoadhesive administration forms for administering cannabis agents - Google Patents n.d.)	Delivery mode	LTS LOHMANN THERAPIE-SYSTEME AG	1991	64
US5227537 Method for the production of 6,12-dihydro-6-hydroxy-cannabidiol and the use thereof for the production of trans-delta-9-tetrahydrocannabinol (US5227537A - Method for the production of 6,12-dihydro-6-hydroxy-cannabidiol and the use thereof for the production of trans-delta-9-tetrahydrocannabinol - Google Patents n.d.)	Production of synthetic cannabinoid	HEINRICH MACK NACHF.	1993	61
WO2017026897A1 Extraction device and an extraction method for extraction of cannabis (WO2017026897A1 - Extraction device and an extraction method for extraction of cannabis - Google Patents n.d.)	Extraction	BRUINING, WERNARD ERNEST	2015	4

The authors have researched the most cited patents wielded by patent examiners against patent applications in the downstream category. Some of the most cited patents presented in Table 7 are not necessarily from downstream categories themselves, yet have been cited by the examiner during examination. It is often the case that, quite legitimately, the examiner will cite from any document felt to have a bearing on patentability even if the citations were not from the same patent category. It can be observed that, in the downstream medical cannabis arena, the data shows that most of the key cited patents have expired. Therefore, while the expired patents noted in Table 7 do not restrict freedom to operate, they may hamper patentability due to prior art issues (novelty or inventiveness).

The technology fields are still rather open to patent competition. Within the top patent holders, and the newly formed companies, there are no great conglomerates of patents. This bodes well for researchers and investors alike who may see opportunities, given the patent filing trends shown in the figures.

A useful way of classifying technology fields within medical cannabis is to use the WIPO authorized International Patent Classification (IPC) hierarchical system. The IPC provides language-independent symbols to designate patented technology within a field. This tool has been used to generate Table 8, showing the number of patents accumulated in each designated technology area within the domain of medical cannabis. It can be seen that there are over 250 patents directed towards preparations and carriers (the A61K classified groups) and approximately 150 patents directed towards various medical conditions and indications (the A61P classified groups).

**Table 8:** Technology areas found in medical cannabis patent survey

IPC	Subject	Number of patent documents
A61K31	Medicinal preparations	100
A61K36	Medicinal preparations form undetermined veg. matter or traditional herbs	78
A61K45	Medical, dental preparations bandages dressings	33
A61K9	Medical, dental preparations in special form	35
A61P17	Dermatological	19
A61P35	Cancer, antineoplastic	17
A61K8	Cosmetic, skin care	13
A61P25	Disorders of the nervous system	78
A61P29	Anti-inflammatory, analgesic	21
A61K47	Medicinal form, carriers	35

In spite of the large number of patentable innovations in various disease conditions as reported above, disappointingly, few cannabinoid drugs have been granted marketing approval by authorities such as the Food and Drug Administration (FDA) or the European Medicines Agency (EMA). These approved drugs are presented in Table 9.

**Table 9:** Approved cannabis-based drugs by a national regulatory authority for clinical use and related patent data

Drug proprietary name	Active pharmaceutical ingredient	Medical condition	Company	Country and year of approval	Relevant patents and dates of expiry
Cesamet	Nabilone (synthetic cannabinoid)	Nausea, Multiple sclerosis, Fibromyalgia	Eli Lilly, Valeant	USA, 1985,2006, UK, EU Austria, Belgium, Spain, variously from 2007	US4195078—expired 1999 EP2004075590—expired 2018
Epidiolex	Cannabidiol (CBD)	Epilepsy	GW Pharma	USA 2018 EU 2019	US10/468041—expiry date 2026 US9956185—expiry date 2035 US9066920—expiry date 2032
Marinol	Dronabinol (synthetic Tetrahydrocannabinol (THC)	HIV/AIDS-induced anorexia and chemotherapy-induced nausea and vomiting	Solvay Pharmaceuticals	USA 1985, 1992	US5166145—expired 2010 US6383513—expired 2018
Sativex	THC and CBD in a 1:1 ratio	neuropathic pain, spasticity, overactive bladder, and other symptoms of multiple sclerosis	GW Pharma	UK 2010 and various European countries, Canada 2005	US20060135599A1—expiry date 2022 US10538373—estimated expiry date 2022
Syndros	Synthetic THC (Dronabinol) in liquid formulation for oral administration	Nausea and vomiting caused by chemotherapy, loss of appetite	Insys Therapeutics	US 2016	US9345771—expiry date 2028 US8222292—expiry date 2028

### Exemptions in patent law affecting research in medical cannabis

Medical cannabis research is a very often applied research with commercialization as the ultimate goal. Therefore, it is essential for MC researchers to consider the potential of patent law to block a research program whether the researchers are affiliated to academia, research institutions, companies, or collaborations.

The exclusive rights conferred to the owner of a patent are wide in scope to incentivize investment in useful technological advance for the benefit of society as a whole. Comprehensive enforcement of these IPR without exception may not always serve the best interests of the public. A need to balance between the rights of the patent owner and the broader interests of society is recognized by most national governments. In national jurisdictions and regional authorities, laws, regulations, and practices are enacted to provide exceptions and safe harbors to the maximal patent exclusivity right (Experts’ Study on Exclusions from Patentable Subject Matter and Exceptions and Limitations to the Rights n.d.). Among these exceptions are (i) private and/or non-commercial use, (ii) experimental use and/or scientific research, (iii) extemporaneous preparation of medicines, (iv) prior use, (vi) acts for obtaining regulatory approval from authorities, (vii) exhaustion of patent rights, (viii) compulsory licensing and/or government use, and (ix) certain use of patented inventions by farmers and breeders.

Likely to be pertinent to the MC researchers are research exemptions or safe harbor exemptions to the rights conferred by patents, particularly in the case of drugs where regulatory or marketing approval is sought. In the USA, these exemptions are called the § 271(e)(1) exemptions or Hatch-Waxman exemptions, but in many countries they are referred to as Bolar provisions (Roche Products, Inc. Appellant, v. Bolar Pharmaceutical Co., Inc., Appellee, 733 F.2d 858 (Fed. Cir. 1984) : Justia n.d.).

Many nations have similar exemptions in place of varying nature and scope. For example, India clearly and broadly provides Bolar type exemptions for research under Section 107A of the Indian Patent Act.

In the European Union, Bolar equivalent exemptions are allowed under the terms of EC Directives 2001/82/EC (as amended by Directive 2004/28/EC) and 2001/83/EC (as amended by Directives 2002/98/EC, 2003/63/EC, 2004/24/EC and 2004/27/EC).

A comparative study of patent law (Jaenichen and Pitz 2015) determined that Great Britain, France, Spain, Italy, Germany, and the Netherlands do not extend patent rights to studies and trials necessary for obtaining drug approval for the marketing of a drug. In Great Britain, it has not been settled whether the patented product used as a tool during a process for obtaining regulatory approval is exempt.

In the USA, exemptions from patent enforcement rights exist when the research using the patented product is done in order to obtain regulatory approvals for use, manufacturing, or marketing approvals (Russo and Johnson 2015).

In Eli Lilly and Co. v. Medtronic, 496 US 661 (1990) (Lilly, and Co. v. Medtronic, Inc. :: 496 U.S. 661 (1990) :Justia US Supreme Court Center n.d.), the US Supreme Court held that the exemption also applies to medical devices, an interesting decision that affects cannabis delivery devices.

University researchers should also note well that experimentation by universities and not-for-profit-organizations using patented products may not be exempt even if the intention is not directly commercial. The university’s experiments may result in publications advancing the prestige of the university or its researchers, possibly attracting funds and students, which constitutes furtherance of a business activity. As a consequence, the research is prohibited (Cai 2004).

In light of the above, researchers in medical cannabis should note that patent law allows sufficient room for research activities to promote innovation (Jaenichen and Pitz 2015), but academic researchers must be aware of potential pitfalls, particularly in light of the restrictions to ostensibly academic research at research institutes in the USA.

### Medical cannabis patent litigation and freedom to operate

To date, there has been little litigation concerning patent infringement, probably because of relatively recent acquisition of IPR concerning MC research, but this trend is likely to change. Patented CRISPR technology is increasingly used to edit plant genes without inserting foreign genetic material. Patents for gene editing in plants are an established feature of the plant innovation landscape. These gene editing patents often cover a wide variety of plants or are general to all plants including Cannabis. Two examples of patents potentially limiting to gene editing in Cannabis are further detailed in Table 10.

**Table 10:** Examples of patents potentially limiting to cannabis gene editing

Title	Short description	Patent doc. number
Methods and compositions for targeted gene modification (US9957515B2 - Methods and compositions for targeted gene modification - Google Patents n.d.)	Methods and compositions for effecting a targeted genetic change in DNA in a cell. The invention relates to improving the efficiency of the targeting of modifications to specific locations in genomic or other nucleotide sequences	US9957515
Methods and compositions for increasing efficiency of targeted gene modification using oligonucleotide-mediated gene repair (US10287594B2 - Methods and compositions for increasing efficiency of targeted gene modification using oligonucleotide-mediated gene repair - Google Patents n.d.)	Improved methods for the modification of genes in plant cells, and plants and seeds derived therefrom. The invention relates to the increased efficiency of targeted gene mutation by combining gene repair oligonucleotides	US10287594

US patent 9957515 to Cibus claims use of a gene repair oligonucleotide or “GRON” when introduced into any plant cell when used with a CRISPR. Another CIBUS patent, US10287594, covers use of the GRON in combination with other strand breaking gene editing elements as well as CRISPR. When contemplating using gene editing, it is advisable for applied researchers in MC to be well acquainted with such patents that may block or limit their patentability in MC or limit the freedom to operate of an applied MC researcher.

It is clear from the presented patent data that much of the patenting activity has been focused on the downstream product segment. It is in this segment that the first patent litigation conflicts have arisen. Such litigation signals clearly to the MC researchers that patent holders value patents in this field and, through the agency of patent law, are willing to prevent competitor researchers from utilizing patented technology without consent of the patent holder. Patents are also opposed by competitors to increase the freedom of their MC researchers to operate.

The most notable cases so far are the GW pharmaceuticals case litigated at the United States Patent and Trademark Office (USPTO) and the United Cannabis Corporation Case, which was litigated at the United States District Court for the District of Colorado (see Table 11).

**Table 11:** Patent litigation cases in medical cannabis field

Title	Patent owner	Alleged Infringer	Patent doc. number
Use of one or a combination of phyto-cannabinoids in the treatment of epilepsy	GW Pharma Ltd.	INSYS Therapeutics	US9066920 (US9066920B2 - Use of one or a combination of phyto-cannabinoids in the treatment of epilepsy - Google Patents n.d.)
Cannabis extracts and methods of preparing and using same	United Cannabis Corp.	Pure Hemp Collective	US9730911 (US9730911B2 - Cannabis extracts and methods of preparing and using same - Google Patents n.d.)

#### GW Pharmaceuticals v. INSYS Therapeutics

In this case, US patent No. US9066920 (US9066920B2 - Use of one or a combination of phyto-cannabinoids in the treatment of epilepsy - Google Patents n.d.) belonging to GW Pharmaceuticals was subject to a patentability challenge by INSYS Therapeutics. This patent is one that potentially protects GW’s FDA approved Epidiolex (CBD) for epilepsy.

The grounds for invalidation asserted by INSYS before the USPTO Patent Trial and Appeal Board (PTAB) were essentially obviousness. INSYS complained that previous publications provided sufficient guidance to an expert in the field in order to obtain the claimed invention, namely that CBD of plant origin of a given dosage was effective against certain types of epilepsy and that this was therefore too obvious to deserve patent protection. Although claims 1 and 2 were invalidated in the proceedings, the PTAB found the essence of the patent (recited in the remaining claims) to be sound and thus assertable against an infringer.

The authors comment that this case demonstrates the importance of obtaining a patent as part of an overall business strategy. Although it took about 6 years for GW pharmaceuticals to obtain the patent and FDA approval was only granted in 2018, the patent will be valid for another 12 years at time of writing, providing GW with monopoly rights. The first external challenge to the patent was beaten off such that interested parties will comprehend the value of such a patent in a medical cannabis IP and product portfolio.

#### UCANN v. Pure Hemp Collective

Another example heralding the importance of patents in this high-value area is the case of United Cannabis Corporation (“UCANN”), which, in 2018, sued Pure Hemp Collective Inc. (“Pure Hemp”) in the United States District Court for the District of Colorado (*United Cannabis Corporation v. Pure Hemp Collective, Inc.* (Case No.: 1:18-cv-01922-NYW) (United Cannabis Corporation v. Pure Hemp Collective Inc., 1:18-cv-01922 n.d.) for patent infringement. UCANN found a similar product allegedly within the scope of their granted claims in terms of concentrations. Here, the defendant Pure Hemp asserted that the specification of UCANN’s patent was not sufficiently clear as to define the boundary of the claim. Judge Martinez then directed the parties to obtain a summary judgment as to the indefiniteness issue in the claims. At the time of writing, the issue has not been settled but few can doubt that it was important for UCANN to file the patent. At the very least, the contentiousness of the patent and court proceedings must be expensively distracting to UCANN’s competitor while UCANN gains traction in the market. These two cases illustrate the value to researchers of obtaining a more than superficial knowledge of patents in the MC field.

### Indigenous Property Rights, traditional knowledge, and cannabis IPRs

The Scottish physician W.B. O’Shaunghnessy can be said to have introduced cannabis to Western medicine in 1841 after observing its use in India and carrying out animal trials prior to treating patients. In cultures and countries as varied as China, India, Zimbabwe, South Africa, Brazil, and Jamaica, traditional medical uses of cannabis include menstrual fatigue, gout, rheumatism, constipation, anesthesia, bronchitis, asthma, diabetes, anthrax, malaria, blackwater fever, blood poisoning, dysentery, glaucoma, diarrhea, fever, burns, abrasions, and wasting disease. It can be thus said that the contributions of modern researchers are, to an extent, built upon contributions of traditional non-western cultures and their ancestors (Spicer 2002).

The data presented in the previous sections attests to the very recent commercial medicalization of cannabis, but medical and therapeutic cannabis has a very long history. Indigenous and local peoples of many countries and regions have been preparing traditional cannabis remedies and selecting and stewarding local landraces, cultivars, and varieties of cannabis plants for centuries (Duvall 2016). Geographically and culturally isolated gene pools were blended in unprecedented combinations to develop both industrial hemp and recreational/medicinal Cannabis cultivars that would be productive when grown in environments to which they were adapted. Human selection has increased seed and fiber yield and quality as well as altered cannabinoid profiles.

Local and indigenous farmers, as well as communities, in developing and emerging regions that traditionally grew Cannabis have valued it in their cultures and made the herb popular in the first place. These farmers must be safeguarded against being excluded from rewards and economic opportunities now flowing towards the burgeoning developed world industrialization of medical cannabis. The above-mentioned conventional IPR (patents and PBR) work in favor of modern-day research in the developed world and offer little or no protection to the indigenous stakeholders. Examples of plants long known by indigenous peoples and nurtured and stewarded by them whose natural qualities are not guaranteed by patent law are shown in Table 12. These examples reveal that the patent system is inadequate for ensuring rights to Indigenous Peoples. Accordingly, it would be desirable to protect the rights of these peoples in the rush to patent medical cannabis research. There is a growing demand for industry to share some of its rewards with the traditional knowledge custodians and practitioners that provided the building blocks of medical cannabis knowledge to the medical cannabis researcher. What is being done to ensure that traditional knowledge (TK) owners will be rewarded for their stewardship, nurturing, and knowledge, passed down through the generations? Commercial breeders may use traditional farmers as a source of cannabis to produce advantageous hybrids, which then may be protected by Plant patents or PBR. Other commercially useful patent-protected cultivation methods or extraction methods or medical uses may have partial origins in TK of a particular Indigenous People.

**Table 12:** Patents concerning plants known to Indigenous Peoples

Plant	Title	Assignee	Patent doc. number
Oubli (*Pentadiplandra brazzeana*	Brazzein sweetener (US5326580A - Brazzein sweetener - Google Patents n.d.)	Wisconsin Alumni Res.Found.	US5326580
*Azadirachta indica* (Neem)	Fungicidal compositions derived from neem oil and neem wax fractions (US5409708A - Fungicidal compositions derived from neem oil and neem wax fractions - Google Patents n.d.)	W.R. Grace	US5409708A
*Hoodia Plant Hoodia gordonii*	Pharmaceutical composition having appetite suppressant activity (US6376657B1 - Pharmaceutical compositions having appetite suppressant activity - Google Patents n.d.)	Council of Scientific and Industrial Research	US6376657
*Hoodia Plant Hoodia gordonii*	Processes for production of Hoodia plant extracts containing steroidal glycosides (WO2008019920A1 - Processes for production of hoodia plant extracts containing steroidal glycosides - Google Patents n.d.)	Unilever	WO2008019920A1

In order to provide resolution platforms for these matters, there are some internationally agreed legal instruments available; the 2014 Nagoya protocol is a supplementary agreement to the Convention on Biological Diversity (CBDR) of 1992 (Smith et al. 2018). It provides a means by which fair distribution of benefit and fair access can be allotted to Indigenous Peoples and other custodians of traditional knowledge and germplasm. Although not an IPR for rewarding innovation as such, rights conferred by CBDR/Nagoya fulfil an IPR function for the custodianship and curation of important plant species that are so fundamental to all agriculture, including *C. Sativa*. There is every reason to expect that such a “quasi IPR” will be relevant to cannabis since obligations under the Nagoya Protocol apply to the use of genetic resources and traditional knowledge. Uses of resources and TK are defined as research, development, innovation, pre-commercialization, or commercialization as well as the benefits arising from their utilization. Contracting parties are obliged to take measures in relation to access to genetic resources, benefit-sharing, and compliance.

The protocol includes prior informed consent (PIC) procedures between traditional knowledge providers and companies, mutually agreed terms (MAT), access permits, and benefit-sharing obligations, whether monetary or non-monetary (royalties and the sharing of research data). These obligations are harmonized with the domestic legislation or regulatory requirements of the contracting party providing genetic resources. A permit is issued when access is granted by administration of the Access and Benefit Sharing Clearing House (ABCH). Compliance is acknowledged by an Internationally Recognized Certificates of Compliance (IRCC) as voucher evidence that the accessed genetic resources or traditional knowledge have appropriate PIC and MAT in accordance with the Access and Benefit Sharing (ABS) requirements of the provider country.

It is not difficult to foresee that the evolution of instruments such as the Nagoya Protocol with future modifications will have a serious role to play in the multinational and multicultural relationships that are forming around medical cannabis commercialization and IPR rights. The authors foresee that research organizations, institutions, and companies with a stake in medical cannabis will incorporate compliance with the Nagoya Protocol and similar ABS regulations into the standard IPR due diligence process for commercial risk mitigation before committing resources into research and development of medical cannabis products.

## Conclusions

In this review of IPR data, we have presented characteristics of intellectual property landscape of the medical cannabis field. IPR data is distributed along the entire MC supply chain, following the upstream–midstream–downstream paradigm.

Agri-technology specific to the cultivation of cannabis plants is not well covered, opening up much scope for research in this area. Work has been reported on genetic manipulation of the cannabis plant, which is likely to have an impact on the discourse framed around MC as a natural herbal therapeutic. Patents directed to gene editing in cannabis are making their appearance in the literature. It is likely that researchers in this field will have to be wary of the contentiousness of patent holders of CRISPR/Cas9 system patents in their attempt to prevent freedom to operate blocks to their research.

In contrast, traditional variety development has led to over 300 protected cannabis varieties according to observed PBR and Plant Patent data. It is to be expected that more variety registrations and plant patents will be granted in the USA as a result of continuing legislative changes easing the path of this branch of MC research.

Remote monitoring and analysis of cannabinoid content, biological control of cannabis plants, successful endogenous, and exogenous expression of cannabinoids in cannabis plant cells have not been convincingly reported although progress has been made using yeast and other cell expression systems. Biosynthetic production of cannabinoids by exploiting enzymes of the cannabinoid metabolic pathways has been reported, with several extraction methods having been claimed. These too are all areas that certainly warrant further research.

Although IPR (especially patents) in the upstream and midstream technologies are not as numerous as other equivalent fields in agritech, approximately ten-fold more patents have been filed in the downstream therapeutic sector. Medical cannabis products cannot be successfully and efficiently generated without full knowledge and development of upstream–midstream processes required to provide characterization and consistency of the downstream product or drug. This may well explain why, although a large group of patent families exist encompassing various medical indications, only three cannabis derived drugs, Epidiolex, Sativex, and synthetic Syndros (liquid formulation of THC), have received FDA marketing approval. The broad implications to downstream research are that upstream and midstream research must be bolstered and the number, quality, and depth of clinical trials and clinical research should be increased. The patent review data supports the notion that multidisciplinary discourse between researchers along the entire supply chain is likely to yield fruitful results.

There is much opportunity for research but researchers should be aware of the potential risks and challenges that patents pose to inventors. Two contentious patent cases are reported, which no doubt herald future infringement and invalidation challenges to claimed IPR in this industry. Such cases are important since they reinforce to researchers the importance of obtaining and defending patents and maintaining hard won priority advantages.

The authors predict that IPR scenarios in medical cannabis will be further enriched by the emergence of international and national “quasi – IPR” in the form of the CBDR, Nagoya protocol, and other arrangements to facilitate research partnerships between multidisciplinary departments in academia, industry, emerging nations, and local and Indigenous Peoples.

Our review provides evidence that an understanding of the pertinent IPR environment is essential for MC researchers to (i) minimize risks of having their work hindered, halted, or made irrelevant and (ii) recognize research opportunities of unfulfilled needs.

## Data Availability

The datasets used and analyzed in this review article are available from the corresponding author upon reasonable request.

## Abbreviations

ABCH: Access and Benefit Sharing Clearing House
ABS: Access and Benefit Sharing
CBD: Cannabidiol
CBDR: Convention on Biological Diversity
CPVO: Community Plant Variety Office
EMA: European Medicines Agency
FDA: Food and Drug Administration
IRCC: Internationally Recognized Certificates of Compliance
IP: Intellectual Property
IPC: International Patent Classification
IPR: Intellectual Property Rights
MAT: Mutually agreed terms
MC: Medical cannabis
NIH: National Institutes of Health
PBR: Plant breeders’ rights
PCT: Patent Cooperation Treaty
PIC: Prior informed consent
PTAB: Patent Trial and Appeal Board
PVP: Plant variety protection
PVPR: Plant Variety Protection Rights
THC: Delta-9-tetrahydrocannabinol
TRIPS: Trade-Related Aspects of Intellectual Property Rights
UPOV: International Union for the Protection of New Varieties of Plants
USPTO: United States Patent and Trademark Office
WTO: World Trade Organization
